# Effect of traditional Chinese medicine emotional therapy on post-stroke depression: A protocol for systematic review and meta-analysis

**DOI:** 10.1097/MD.0000000000025386

**Published:** 2021-04-09

**Authors:** Yanhong Yang, Ming Zhang, Junjie Zhao, Shanshan Song, Feifei Hong, Guiqin Zhang

**Affiliations:** Jinan People's Hospital Affiliated to Shandong First Medical University, Jinan, Shandong Province, China.

**Keywords:** depression, nursing, protocol, stroke, systematic review

## Abstract

**Background:**

Post-stroke depression has seriously affected the rehabilitation and quality of life of patients, and there is no reliable treatment plan at present. Nursing plays an important role in the recovery of patients, some studies have pointed out that traditional Chinese medicine emotional therapy has advantages in improving post-stroke depression and promoting rehabilitation, but it is lack of evidence-based basis. The purpose of this study is to systematically evaluate the effect of traditional Chinese medicine emotional therapy on the improvement of post-stroke depression.

**Method:**

We will search CNKI, Wanfang, VIP and CBM, PubMed, Embase, Web of Science and the Cochrane Library database, and search the randomized controlled trial on traditional Chinese medicine emotional therapy in patients with post-stroke depression from the establishment of the database to February 2021. The language is limited to English and Chinese. The quality of the included study is independently extracted and the literature quality is evaluated by 2 researchers. And meta-analysis is performed on the included literature using RevMan5.3 software.

**Result:**

In this study, the effect of traditional Chinese medicine emotional therapy on the improvement of post-stroke depression is evaluated by patient psychiatric scale score, compliance evaluation, quality of life evaluation and other indicators.

**Conclusion:**

This study will provide reliable evidence-based basis for establishing a reasonable and effective nursing scheme for patients with post-stroke depression.

**Ethics and dissemination:**

Private information from individuals will not be published. This systematic review also does not involve endangering participant rights. Ethical approval will not be required. The results may be published in a peer-reviewed journal or disseminated at relevant conferences.

**OSF Registration number:**

DOI 10.17605/OSF.IO/KA7G3

## 1 Introduction

Stroke is one of the leading causes of death and disability in the world.^[1]^ Stroke is the second most common cause of death in China.^[2]^ A large number of stroke patients face serious physical defects and emotional disorders dominated by anxiety and depression.^[3]^ Some studies have pointed out that the prevalence rate of post-stroke anxiety is 20% to 25%,^[4]^ and the cumulative incidence of post-stroke depression is 55%,^[5]^ which seriously affects the prognosis and quality of life of stroke patients.^[6]^ This state is mainly manifested in treatment compliance, such as patients’ lack of confidence in treatment and prognosis, failure to actively follow the doctor's advice to take medicine and rehabilitation training, which not only affects the treatment and rehabilitation of the disease. At the same time, it also causes a great burden to the society, the family, and even the patients themselves.^[7]^

At present, the improvement of post-stroke depression includes intervention measures such as drugs, psychological or alternative therapy.^[8]^ In recent years, traditional Chinese medicine emotional therapy has also been widely used in the intervention of negative emotion after stroke. Traditional Chinese medicine emotional therapy, also known as traditional Chinese medicine emotional nursing, under the guidance of the basic theory of traditional Chinese medicine, through understanding the emotional changes of patients and grasping their psychological state, nurses influence patients through speech, attitude, expression, behavior, temperament, and other aspects. To achieve the purpose of regulating the overall cognition, healthy feeling, daily life, and improving the negative mood of depression, so as to improve the confidence of patients to overcome the disease. Receive treatment and nursing in a good mental state.^[9]^

At present, a number of randomized controlled studies have confirmed that emotional nursing can reduce the level of depression in patients with post-stroke depression and play a positive role in promoting the recovery of neurological function and limb motor function.^[10–14]^ However, due to the differences in research programs and nursing contents, there are differences in research results and lack of reliable evidence-based basis, which limits the promotion of this program. Therefore, this study planned the effect of systematic traditional Chinese medicine emotional therapy on the depressive state of stroke patients, to get a standardized and reliable conclusion.

## 2 Methods

### 2.1 Protocol register

This protocol of systematic review and meta-analysis has been drafted under the guidance of the preferred reporting items for systematic reviews and meta-analyses protocols (PRISMA-P).^[15]^ Moreover, it has been registered on open science framework (OSF) (Registration number: DOI 10.17605/OSF.IO/KA7G3).

### 2.2 Ethics

Since the programme does not require the recruitment of patients and the collection of personal information, it does not require the approval of the Ethics Committee.

### 2.3 Inclusion criteria

#### 2.3.1 Type of studies

This study will include a randomized controlled study using traditional Chinese medicine emotional nursing to manage patients with post-stroke depression.

#### 2.3.2 Type of participants

Patients included in the study were diagnosed with post-stroke depression, aged >18 years, regardless of nationality, age, sex, or stroke type.

The observation group was treated with traditional Chinese medicine emotional nursing, whereas the control group was treated with traditional nursing (the form of nursing was not limited).

#### 2.3.3 Types of outcome measures

Main Outcome Measures: psychiatric scale score, including Self-rating Anxiety Scale, Self-rating Depression Scale, Hamilton Anxiety Scale, Hamilton Depression Scale, Symptom Checklist-90 Scale, and so on.

Secondary outcome indicators were: compliance evaluation, such as medication compliance, functional training compliance, and so on; quality of life evaluation, such as the Mos 36-item Short Form Health Survey (SF-36), Activity of Daily Living Scale (ADL), and so on.

### 2.4 Exclusion criteria

1. The published literature is abstract, review and animal research.
2. Repeatedly published literature for the same research population.
3. Articles with incomplete or incorrect data and unable to obtain complete data after contacting the author.
4. Literature with inconsistent intervention methods or no related outcome indicators.

### 2.5 Retrieval strategy

With “stroke,” “cerebral infarction,” “hemiplegia,” “ traditional Chinese medicine emotional nursing,” and “ traditional Chinese medicine emotional therapy” as the Chinese search terms, in CNKI, Wanfang, VIP and CBM, while “traditional Chinese medicine emotional nursing,” “traditional Chinese medicine emotional therapy,” “TCM emotional therapy,” “stroke,” “emotional disorder,” and “depression” are searched in PubMed, Embase, Web of Science, and the Cochrane Library. The retrieval time is of the establishment of the database in February 2021, and all the literature on the application of emotional nursing in post-stroke rehabilitation is collected. Take PubMed as an example, the retrieval strategy is shown in Table 1.

**Table 1:** Search strategy in PubMed database.

Number	Search terms
#1	traditional Chinese emotional nursing [Title/Abstract]
#2	traditional Chinese emotional therapy [Title/Abstract]
#3	TCM emotional nursing [Title/Abstract]
#4	TCM emotional therapy [Title/Abstract]
#5	#1 OR #2 OR #3 OR #4
#6	Stroke [MeSH]
#7	Cerebrovascular Accident [Title/Abstract]
#8	Stroke, Cerebrovascular [Title/Abstract]
#9	Brain Vascular Accident [Title/Abstract]
#10	Acute Cerebrovascular Accident [Title/Abstract]
#11	Acute ischemic stroke [Title/Abstract]
#12	Cerebrovascular Accidents, Acute [Title/Abstract]
#13	Cerebrovascular Apoplexy [Title/Abstract]
#14	#6 OR #7 OR #8 OR #9 OR #10 OR #11 OR #12 OR#13
#15	Depression [MeSH]
#16	Depressive Symptom [Title/Abstract]
#17	Emotional Depression [Title/Abstract]
#18	Emotional disorder [Title/Abstract]
#19	#15 OR #16 OR #17 OR #18
#20	#5 AND #14 AND #19

### 2.6 Data screening and extraction

**Figure 1:**
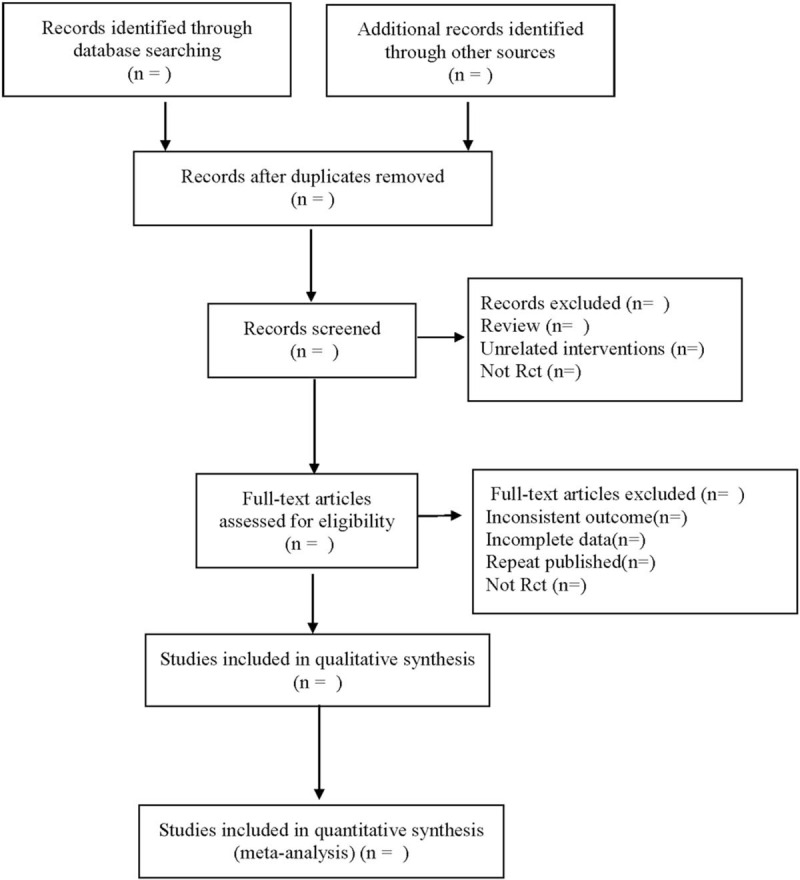
Flow diagram.

The data will be extracted independently by the 2 researchers, the information will be recorded in the data extraction form, and their differences will be resolved with the help of a third reviewer. The detailed information is extracted as follows: ① clinical study (title, first author, year and year of publication, sample size, sex ratio, average age, length of stay, stroke type); ② intervention measures (nursing type, nursing plan, duration, follow-up time); ③ risk bias assessment factors in randomized controlled trials; ④ outcome indicators. The literature screening process is shown in Figure 1.

### 2.7 Literature quality assessment

The quality evaluation of the literature is completed independently by two researchers according to the Cochrane5.1.0 system evaluation manual. When there is disagreement, the third researcher participates in the discussion, and finally decides the overall quality of the literature. Evaluation indicators include random sequence generation, distribution hiding, blind method, integrity of result data, selective reporting of research results, and other sources of bias. According to these indicators, the included literature is evaluated as “high bias risk,” “low bias risk,” and “unknown.”

### 2.8 Statistical analysis

#### 2.8.1 Data analysis and processing

RevMan 5.3 software is used for meta-analysis. The heterogeneity of each literature is judged by testing and *I*^*2*^, and the heterogeneity of the effect value is analyzed. If *P* *>* .1*, I*^*2*^* <* 50%, it shows that the heterogeneity among the studies is low, so a fixed model is used for analysis; if *P* *<* .1*, I*^*2*^* ≥* 50%, there is obvious heterogeneity among the studies. The sources of heterogeneity are analyzed and the random effect model is used for analysis. The measurement data are expressed as the weighted mean difference or the standard mean difference and 95% CI, and the counting data are expressed as relative ratio (RR) and 95% CI.

#### 2.8.2 Dealing with missing data

If the data of the required study are incomplete or not reported in the study, the researcher will contact the first author or other author of the study by phone or email. If the required data are not available, we will use descriptive analysis instead of meta-analysis and exclude these studies if necessary.

#### 2.8.3 Subgroup analysis

We will further explore the effect of emotional nursing on patients with post-stroke depression according to stroke type, nursing plan, age of patients, and other factors.

#### 2.8.4 Sensitivity analysis

To test the stability of the meta-analysis results of each index, we will use the one-by-one elimination method to analyze the sensitivity of the main outcome indicators.

#### 2.8.5 Assessment of reporting biases

If there are >10 studies, funnel chart is used to evaluate whether there is publication bias. Moreover, Egger and Begg test are used for the evaluation of potential publication bias.

#### 2.8.6 Evidence quality evaluation

We will use the Grading of Recommendation Assessment, Development and Evaluation (GRADE) scoring method to grade the evidence of the outcome index.^[16]^ The evaluation content includes bias risk, indirectness, inconsistency, inaccuracy and publication bias, and the quality of evidence will be rated as high, medium, low, or very low.

## 3 Discussion

The depression of stroke patients is characterized by persistent depression and loss of interest, which plays a reverse role in the treatment and rehabilitation of stroke.^[17]^ If not treated in time, the occurrence of depression can delay the recovery of neurological function, or even aggravate physical disorders,^[18]^ serious cases can lead to suicide, bring great pain and burden to patients and their families.^[19]^ The efficacy of antidepressants in the treatment of post-stroke depression is not only limited, but also has side effects, and the compliance of patients is poor.^[13]^

Patients with post-stroke depression are resistant to external stimuli, and their treatment and clinical nursing are difficult, so they need to pay more attention. Traditional Chinese medicine emotional therapy is a systematic nursing scheme based on 7 emotions (joy, anger, worry, thought, sadness, fear, surprise) and 5 emotions (anger, joy, thought, worry, fear) in the traditional theory of traditional Chinese medicine. Transfer the patient's attention to the disease through good external conditions, urge the patient to maintain a good state of mind and relax from the psychological and spiritual level.^[20]^ Its contents include: reasoning and instruction, obedience to the desire, dispelling emotion, suggestion therapy, a variety of emotional stimulation, and so on.^[21]^ Previous studies have confirmed that traditional Chinese medicine emotional nursing can improve the neurological and cognitive function of patients with post-stroke depression, and reduce anxiety and depression of patients.^[22]^ It has the advantages of economic safety, less adverse reactions, easy to be accepted by patients and conducive to the recovery of the disease.^[23]^ Through this systematic evaluation, we will provide reliable evidence-based basis for the application of traditional Chinese medicine emotional therapy in the rehabilitation of patients with post-stroke depression.

However, this systematic review has some limitations. Due to the type of stroke, the age of patients, the specific content of care and other factors will increase the possibility of heterogeneity; due to the limitations of language retrieval, we will only include Chinese and English literature, and may ignore the research in other languages and regions.

## Author contributions

**Data curation:** Yanhong Yang, Ming Zhang.

**Funding acquisition:** Guiqin Zhang.

**Resources:** Junjie Zhao, Shanshan Song.

**Software:** Feifei Hong, Guiqin Zhang.

**Supervision:** Shanshan Song.

**Writing – original draft:** Yanhong Yang, Ming Zhang.

**Writing – review & editing:** Yanhong Yang, Guiqin Zhang.
